# Supplementary figures and images for: Elucidating Scent and Color Variation in White and Pink-Flowered Hydrangea arborescens ‘Annabelle’ Through Multi-Omics Profiling
Source: Plants (Basel). 2026 Jan 4;15(1):155. doi: 10.3390/plants15010155 (PMC12788147; doi:10.3390/plants15010155)

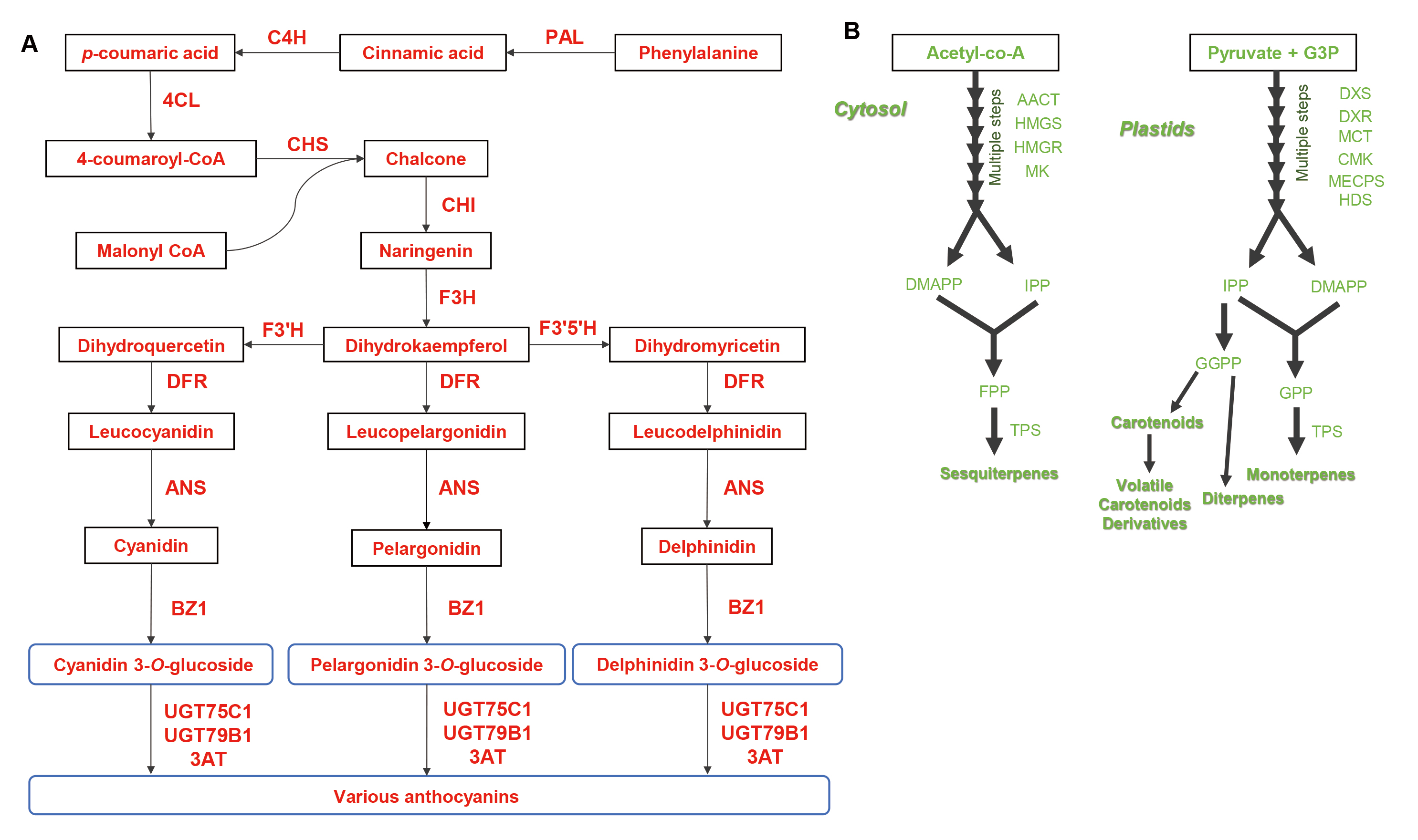

Supplement: Supplementary file 1 [file plants-15-00155-s001.zip › Figure S1.jpg]

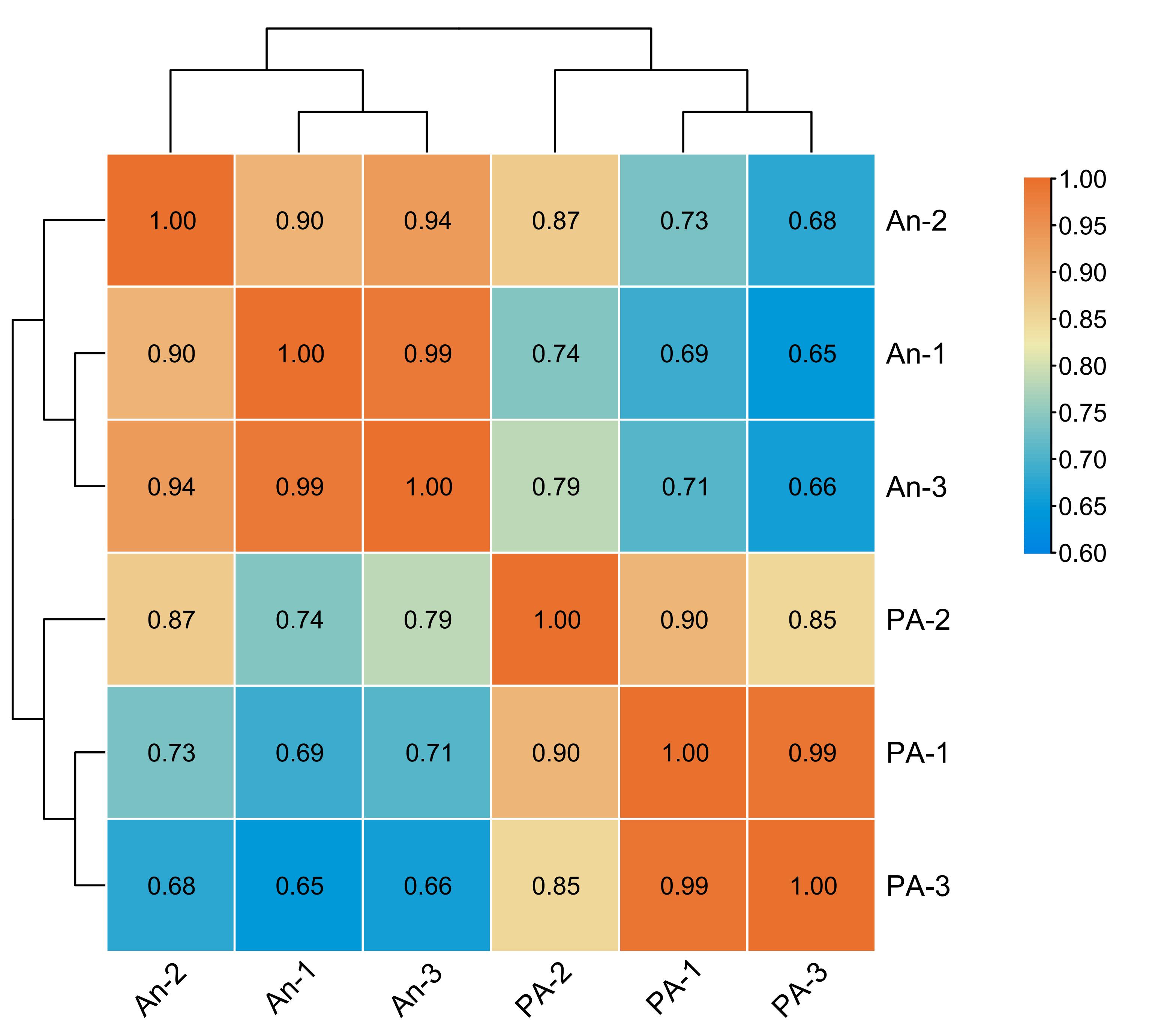

Supplement: Supplementary file 1 [file plants-15-00155-s001.zip › Figure S2.jpg]
